# The role of HER-2/neu expression on the survival of patients with lung cancer: a systematic review of the literature

**DOI:** 10.1038/sj.bjc.6601252

**Published:** 2003-09-09

**Authors:** A-P Meert, B Martin, M Paesmans, T Berghmans, C Mascaux, J-M Verdebout, P Delmotte, J-J Lafitte, J-P Sculier

**Affiliations:** 1 Fonds National de la Recherche Scientifique, Bruxelles, Belgium; 2 Département de Médecine Interne et Laboratoire d'Investigation Clinique et d'Oncologie Expérimentale HJ Tagnon, Institut Jules Bordet, 1, rue Héger Bordet, 1000-Bruxelles, Belgium; 3 Data Centre, Institut Jules Bordet, Bruxelles, Belgiuml; 4 Service d'Anatomo-Pathologie, Institut Jules Bordet, Bruxelles, Belgium; 5 Service de Pneumologie et d'Oncologie Thoracique, CHU Calmette, Lille, France

**Keywords:** lung cancer, systematic review, HER-2/neu, p185, c-erbB2, survival, prognostic factor

## Abstract

C-erbB-2 prognostic value for survival in patients with lung cancer remains controversial. We performed a systematic review of the literature to clarify its impact. Studies were identified by an electronic search in order to aggregate the survival results, after a methodological assessment using the scale of the European Lung Cancer Working Party. To be eligible, a study had to deal with c-erbB-2 assessment in lung cancer patients and to analyse survival according to c-erbB-2 expression. In total, 30 studies were eligible: 24 studies dealt with non-small-cell lung carcinoma (NSCLC), five with adenocarcinoma and one study dealt with small-cell carcinoma. In all, 31% of the patients were positive for c-erbB-2. According to c-erbB-2 expression, 13 studies were ‘negative’ (significant detrimental effect on survival), one ‘positive’ (significant survival improvement) and 16 not significant. Significant studies had a better subscore relative to analysis and results report than nonsignificant studies. In total, 86% of the significant studies and only 56% of the nonsignificant studies were evaluable for the meta-analysis. This suggests a possible bias in our aggregated results. For NSCLC, the hazard ratio was 1.55 (95% CI: 1.29–1.86) in favour of tumours that do not express c-erbB-2. In conclusion, the overexpression of c-erbB-2 might be a factor of poor prognosis for survival in NSCLC, but there is a potential bias in favour of the significant studies with an overestimation risk of the magnitude of the true effect of c-erbB-2 overexpression.

Lung carcinoma is the most common malignant neoplasm in the developed world and represents the leading cause of cancer death in Europe and North America. Surgical resection remains the standard of care for patients with early-stage disease. Unfortunately, 70% of all patients have inoperable disease and despite improvements in the detection and treatment of lung cancer, less than 15% are long-term survivors. This poor prognosis can, however, be modulated by characteristics related to the patient or the tumour. Some independent clinical and biological predictors have been identified for predicting survival (Kanters *et al*, 1995; Paesmans and Sculier, 1998): age, performance status, disease stage, serum lactate dehydrogenase level, white blood cell and neutrophil counts.

Currently, increased attention has been focused on new biological parameters: biological substaging using molecular markers in a risk stratification strategy has been proposed (Pastorino *et al*, 1997; D'Amico *et al*, 1999), although their role as a prognostic factor remains unclear. In particular, a number of studies have been performed to assess the prognostic role of tumour-suppressor genes and proto-oncogenes. Among the proto-oncogenes, the epidermal growth factor (EGF) family plays an important role in local tumour growth. This phenomenon requires growth-regulatory proteins such as epidermal growth factor receptor (EGF-R) or proto-oncogene erb-B2. The HER-2/neu dominant gene is localised in normal human cells as a singular copy on the long arm (q21) of chromosome 17. This gene, first identified in a chemically induced rat neuroblastoma (Schechter *et al*, 1984), codes for a 185-kDa receptor-type tyrosine protein kinases (p185 neu or c-erbB-2) similar to EGF-R. This 1255 amino-acid transmembrane glycoprotein is composed of three domains: an extracellular factor-binding domain, a transmembrane domain and an intracellular domain with tyrosine kinase activity. When an EGF-like ligand (there is no known specific ligand for HER-2) binds to a receptor of the EGF-R family (HER-1, 3 or 4), there is a heterodimerisation of this receptor with HER-2. HER-2 is necessary for the regulation of normal cell growth and differentiation, and can be associated with multiple signal transduction pathways (Hung and Lau, 1999). However, amplification of the HER-2 gene leads to overexpression of the receptor, which is linked to the formation of more HER-2 heterodimers and could be implicated in the development of many types of tumours. HER-2/neu is expressed in a wide variety of human epithelial malignancies, including breast, ovary, salivary gland, gastrointestinal tract, prostate, lung, kidney, liver and bladder carcinomas, suggesting that its overexpression could play a critical role in the development and progression of human cancers. The c-erbB-2 protein product, p185, is expressed in 20–30% of non-small-cell lung cancers (NSCLC) and particularly in adenocarcinoma (Tateishi *et al*, 1991; Kern *et al*, 1994). Numerous studies have suggested that HER-2 expression is associated with advanced or metastatic disease and a poor prognosis (Kern *et al*, 1990; Tateishi *et al*, 1991; Harpole *et al*, 1995a), especially when combined with K-ras mutations (Kern *et al*, 1994) or Bcl-2 (Kim *et al*, 1998), whereas others did not (Pfeiffer *et al*, 1996). Such conflicting results can be explained by the low numbers of patients included in the majority of the studies and by the use of different methods to determine HER-2/neu status. Therefore, we performed a systematic review of the literature to assess the prognostic value of HER-2 overexpression on survival in patients with lung cancer.

## MATERIALS AND METHODS

### Publication selection

The eligibility criteria of the articles were as follows: to deal with lung cancer only, to evaluate the association between c-erbB-2 status and survival, to measure c-erbB-2 expression in the tumour and to be published as a full paper in the English or French language literature. Abstracts were excluded from this research because they provided insufficient data to evaluate the methodological quality of the trial and to perform meta-analysis.

The search was performed by consulting the Medline electronic database, using the keywords ‘lung neoplasms’, ‘c-erbB2’, ‘erb2’, ‘erb-2’, ‘Her-2’, ‘Her2’, ‘Her2/neu’, ‘Her-2/neu’, ‘p185’, ‘epithelial growth factor’ and ‘EGF’. Moreover, the bibliographies reported in all the identified studies were used for completion of the trials search. When authors reported, in several publications, the same patient populations, only the most recent or complete study was included in the analysis in order to avoid overlapping between cohorts. The search ended on August 2002.

### Methodological assessment

A team of nine investigators (including six physicians, one pathologist, one biologist and one biostatistician) performed the methodological evaluation of each trial using the European Lung Cancer Working Party (ELCWP) scale. This scoring system was previously described (Steels *et al*, 2001). Each item of the score was quoted using an ordinal scale (possible values 2, 1, 0) by consensus in meetings where at least two-thirds of the investigators needed to be present. The participation of many readers was a guarantee for the correct interpretation of the articles. The overall score assessed several dimensions of methodology, grouped into four main categories: the scientific design, the description of the laboratory methods used to identify the presence of c-erbB-2, the generalisability of the results and the analysis of the study data. Each category had a maximal score of 10 points with an overall maximal theoretical score of 40 points. When an item was not applicable to a study, its value was not taken into account in the total for the category. The final scores were expressed as percentages, higher values reflecting a better methodological quality. Studies included in the systematic review were called ‘eligible’ and those providing sufficient data for the meta-analysis ‘evaluable’.

### Statistical method

A study was considered as significant if the *P*-value for the statistical test comparing survival distributions between the groups with and without c-erbB-2 expression was <0.05. The study was called ‘positive’ when c-erbB-2 expression was identified as a significant favourable prognostic factor for survival. The study was called ‘negative’ if c-erbB-2 expression was associated with a significant detrimental effect on survival. Finally, a study was called ‘not significant’ if no difference between the groups according to c-erbB-2 expression was detected. The association between the quality scores or between a quality score and another continuous variable was measured by the Spearman ranks correlation coefficient. Nonparametric Mann–Whitney tests were conducted to compare the distributions of the quality scores according to the value of a dichotomic variable.

For the quantitative aggregation of survival results, we compared the survival distributions between the two groups (with or without c-erbB-2 overexpression) by the hazard ratio (HR) according to a method that we have previously reported (Meert *et al*, 2002a). For each trial, the HR was estimated by a method depending on the data provided in the publications. The most accurate method consisted of calculating the estimated HR and its standard error from the reported results or to calculate them directly using two of the following parameters: the confidence interval (CI) for the HR, the logrank statistic, its *P*-value or the O−E statistic (difference between the numbers of observed and expected events). If those data were not available, we looked at the total number of events, the number of patients at risk in each group and the logrank statistic or its *P*-value allowing calculation of an approximation of the HR estimate. Finally, if the only available data were in the form of graphical representations of the survival distributions, we extracted from them survival rates at some specified times in order to reconstruct the HR estimate and its variance, with the assumption that the rate of patients censored was constant during the study follow-up (Parmar *et al*, 1998). The individual HR estimates were combined into an overall HR using Peto's method (Yusuf *et al*, 1985) that consisted of using a fixed-effect model assuming homogeneity of the individual true HRs. This assumption was tested by performing *χ*^2^ tests for heterogeneity. If the assumption of homogeneity had to be rejected, we used a random-effects model. By convention, an observed HR>1 implied a worse survival for the group with c-erbB-2 expression. The impact of c-erbB-2 on survival was considered as statistically significant if the 95% CI for the overall HR did not overlap 1.

## RESULTS

### Studies selection and characteristics

A total of 39 studies, published between 1990 and 2002, were found eligible (Kern *et al*, 1990, 1994; Tateishi *et al*, 1991, 1994; Volm *et al*, 1992, 1993a; Harpole *et al*, 1995a, 1995b, 1996; Giatromanolaki *et al*, 1996a, 1996b; Pfeiffer *et al*, 1996; Koukouraki *et al*, 1997, 2000; MacKinnon *et al*, 1997; Pastorino *et al*, 1997; Visscher *et al*, 1997; Yu *et al*, 1997; Graziano *et al*, 1998; Greatens *et al*, 1998; Hsieh *et al*, 1998; Kim *et al*, 1998; Kwiatkowski *et al*, 1998; Nemunaitis *et al*, 1998; Cantero *et al*, 1999, 2000; D'Amico *et al*, 1999; Fu *et al*, 1999; Moldvay *et al*, 2000; Schneider *et al*, 2000; Ardizzoni *et al*, 2001; Brabender *et al*, 2001; Liao *et al*, 2001; Shou *et al*, 2001; Carbognani *et al*, 2002; Han *et al*, 2002; Hirsch *et al*, 2002; Potti *et al*, 2002; Selvaggi *et al*, 2002). Nine of the articles were excluded because identical cohorts of patients were used (studies that were excluded and included were, respectively, references (Cantero *et al*, 1999) and (Cantero *et al*, 2000), (Giatromanolaki *et al*, 1996b; Koukourakis *et al*, 1997, 2000) and (Giatromanolaki *et al*, 1996a), (Harpole *et al*, 1995a, 1995b) and (Harpole *et al*, 1996), (Kern *et al*, 1994) and (Kern *et al*, 1990), (Tateishi *et al*, 1991) and (Tateishi *et al*, 1994), (Volm *et al*, 1992) and (Volm *et al*, 1993a, 1993b).

The total number of patients included in the review was 4582 (range 31–483; median 117). The principal individual characteristics of these different trials are reported in Table 1

**Table 1:** Main characteristics and results of the eligible studies

**Author**	**Year**	**Histology**	**Stage**	**N pts**	**Sample**	**Technique**	**HR estimation**	**Results**
Ardizzoni (Ardizzoni *et al*, 2001)	2001	NSCLC	III–IV	84	Serum	ELISA (+IHC)	Logrank+*n* events	Negative
Brabender (Brabender *et al*, 2001)	2001	NSCLC	I–IIIA	83	Surgery	PCR	Survival curves	Negative
Cantero (Cantero *et al*, 2000)	2000	NSCLC	I–IIIA	102	Surgery	ELISA	HR+CI	Negative
Carbognani (Carbognani *et al*, 2002)	2002	NSCLC	I–IIIA	78	Surgery	IHC	No data	NS
D'Amico (D'Amico *et al*, 1999)	1999	NSCLC	I	408	Surgery	IHC	Logrank+*n* events	Negative
Fu (Fu *et al*, 1999)	1999	NSCLC	I–IIIB	158	Surgery	IHC	*No data*	NS
Giatromanolaki (Giatromanolaki *et al*, 1996a)	1996	NSCLC	I–II	107	Surgery	IHC	Survival curves	NS
Graziano (Graziano *et al*, 1998)	1998	NSCLC	IIIA	47	Mediastinoscopy	IHC	No data	NS
Greatens (Greatens *et al*, 1998)	1998	NSCLC	I–IV	101	Surgery	IHC	No data	Positive
Han (Han *et al*, 2002)	2002	NSCLC	I	85	Surgery	IHC	Survival curves	Negative
Harpole (Harpole *et al*, 1996)	1996	NSCLC	I	275	Surgery	IHC	Logrank+*n* events	Negative
Hirsch (Hirsch *et al*, 2002)	2002	NSCLC	I–IIIA	187	Surgery+biopsies	IHC (+ FISH)	Survival curves	NS
Hsieh (Hsieh *et al*, 1998)	1998	Adenoc	I	42	Surgery	IHC	Logrank+*n* events	Negative
Kern (Kern *et al*, 1990)	1990	NSCLC	I–IV	44	Surgery+biopsies	IHC	HR+CI	Negative
Kim (Kim *et al*, 1998)	1998	NSCLC	I–IV	238	Surgery+biopsies	IHC	HR+CI	NS
Kwiatkowski (Kwiatkowski *et al*, 1998)	1998	NSCLC	I	243	Surgery	IHC	Logrank+*n* events	NS
Liao (Liao *et al*, 2001)	2001	NSCLC	I–IIIA	127	Surgery	IHC	Logrank+*n* events	NS
MacKinnon (MacKinnon *et al*, 1997)	1997	Adenoc	?	162	Surgery	IHC	No data	NS
Moldvay (Moldvay *et al*, 2000)	2000	NSCLC	I–IV	227	Surgery	IHC	Logrank+*n* events	NS
Nemunaitis (Nemunaitis *et al*, 1998)	1998	Adenoc	I–IV	103	Surgery	IHC	No data	NS
Pastorino (Pastorino *et al*, 1997)	1997	NSCLC	I	483	Surgery	IHC	HR+CI	NS
Pfeiffer (Pfeiffer *et al*, 1996)	1996	NSCLC	I–IV	186	Surgery	IHC	Survival curves	NS
Potti (Potti *et al*, 2002)	2002	SCLC	Extensive	193	Biopsies	IHC	Survival curves	Negative
Schneider (Schneider *et al*, 2000)	2000	NSCLC	I–IIIA	103	Surgery	IHC	No data	Negative
Selvaggi (Selvaggi *et al*, 2002)	2002	NSCLC	I–III	130	Surgery	IHC	HR+CI	Negative
Shou (Shou *et al*, 2001)	2001	NSCLC	I–III	111	Surgery	IHC	Survival curves	NS
Tateishi (Tateishi *et al*, 1994)	1994	Adenoc	I–IV	119	Surgery	IHC	Survival curves	Negative
Visscher (Visscher *et al*, 1997)	1997	Adenoc	I–IV	31	Surgery	IHC	No data	NS
Volm (Volm *et al*, 1993a)	1993	NSCLC	I–III	241	Surgery	IHC	No data	NS
Yu (Yu *et al*, 1997)	1997	NSCLC	I–IIIA	116	Surgery	IHC	Survival curves	Negative

NSCLC=non-small-cell lung cancer; SCLC=small-cell lung cancer; Adenoc=adenocarcinoma; N pts=number of patients; IHC=immunohistochemistry; PCR=polymerase chain reaction; FISH=fluorescent *in situ* hybridisation; NS=not significant; ()=technique used to determine c-erbB-2 status but not used for correlation with survival.

. Among them, 24 dealt with NSCLC of all histologic subtypes, five with adenocarcinoma only and one with small-cell carcinoma only. Patients with locoregional disease (stages I–IIIB) were included in 19 studies, patients with advanced disease were included in two studies, eight studies dealt with all stages diseases and, in one, disease stages were not detailed. In all, 27 trials used immunohistochemistry (IHC), two ELISA and one polymerase chain reaction (PCR) to detect c-erbB-2 expression. Different antibodies were used to assess c-erbB-2 expression by IHC. Assessment of c-erbB-2 status was carried out on surgical (24 studies) and/or bronchial biopsies samples (four studies) whereas nodes obtained by mediastinoscopy and sera were used, respectively, each in one study.

### Study results reports

According to c-erbB-2 expression, in NSCLC, 12 studies (1583 patients) were ‘negative’, one was ‘positive’ (101 patients) and 16 were ‘not significant’ (2705 patients) for survival. In the study considering small-cell lung carcinoma (193 patients), c-erbB-2 overexpression tended to be of worse prognosis.

Overall, according to the positivity threshold for c-erbB-2 expression as defined by the study authors, c-erbB-2 was expressed in 31% of the NSCLC patients, in 30% of the patients with adenocarcinoma and in 30% of the SCLC patients.

c-erbB-2 expression was found in 32% of the patients with locoregional disease and in 36% of those with advanced disease.

Evaluability status for the meta-analysis was associated with trial positivity: the rate of positive results was 57% for evaluable trials (12 out of 21) compared to 11% for nonevaluable ones (one out of nine) (*P*=0.11).

### Quality assessment

Before attempting to aggregate the results of the individual trials, a qualitative assessment of each study was performed. The median quality score for the pooled trials was 57.6% (range 37.4–82.6%). The ‘design’ subscore had the lowest value except for the nonevaluable studies where it was the ‘analysis results’ subscore (Table 2

**Table 2:** Quality scores analysis of the eligible studies

**Studies (number)**	**Design (/10)**	**Laboratory method (/10)**	**Generalisability (/10)**	**Results analysis (/10)**	**Global score (%)**
All (30)	5.00	6.42	6.66	5.62	57.58
Evaluable for MA (21)	5.00	6.42	6.66	6.25	61.07
Nonevaluable for MA (9)	5.00	6.42	7.50	3.75	54.34
*P*	0.15	0.94	0.83	**0.02**	0.09
Significant (14)	5.00	6.42	6.66	6.87	61.19
Nonsignificant (16)	4.50	6.07	6.66	5.00	52.64
*P*	0.30	0.26	0.27	**0.03**	**0.03**
Significant studies in MA (12)	5.00	6.42	6.66	7.50	61.19
Nonsignificant studies in MA (9)	5.00	6.42	6.66	6.25	53.18
*P*	0.91	0.47	0.59	0.14	0.22

MA=meta-analysis. Scores are median scores of the studies.

). No statistically significant difference in terms of quality score was observed between evaluable (range 40.5–82.6%) and nonevaluable studies (range 37.4–68.1%) for meta-analysis (median scores: 61.1 *vs* 54.3%, *P*=0.09). The items not adequately described (median score less than 1) were the statistical considerations, the initial work-up of the disease and the number of unassessable samples with description of exclusion causes. A statistically significant difference in terms of methodological score was found between the significant trials (range 47.3–82.6%) and the nonsignificant trials (range 37.4–72.9%) (median overall scores 61.2 *vs* 52.6%, *P*=0.03), but not between the significant and nonsignificant studies evaluable for the meta-analysis (61.2 *vs* 53.2%, *P*=0.22). The difference in terms of quality score between significant and nonsignificant studies is especially noted in the ‘results analysis’ subscore (including the follow-up description, the survival analysis according to the HER-2, the univariate and the multivariate analysis) with a better results description in the significant studies (6.8 out of 10 *vs* 5.0 out of 10; *P*=0.03). There was no significant correlation between quality scores and the date of publications of the studies or with the number of patients included in these studies.

### Meta-analysis

Out of the 14 significant studies, 12 are included in the meta-analysis and nine out of the 16 nonsignificant studies are included in the meta-analysis.

The HRs of the 21 evaluable studies were calculated by one of the three methods reported in the Materials and Methods section. Hazard ratios and 95% CIs were published in five trials. They were approximated from the logrank statistic and the number of events in seven studies. Finally, the HR and its variability had to be extrapolated from the graphical representations of the survival distributions in the nine others.

**Figure 1:**
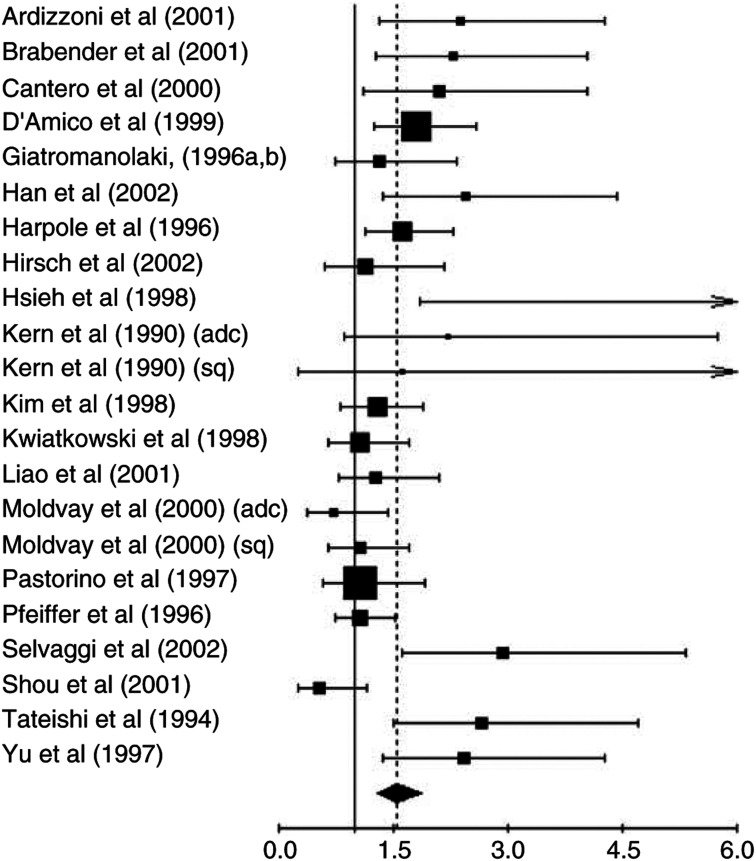
Results of the meta-analysis of all the studies. Ardizzoni *et al* (2001); Brabender *et al* (2001); Cantero *et al*, (2000); D'Amico *et al* (1999); Giatromanolaki *et al* (1996a), (1996b); Han *et al* (2002); Harpole *et al* (1996); Hirsch *et al* (2002); Hsieh *et al* (1998); Kern *et al* (1990) (adc); Kern *et al* (1990) (sq); Kim *et al* (1998); Kwiatkowski *et al* (1998); Liao *et al* (2001); Moldvay *et al* (2000) (adc); Moldvay *et al* (2000) (sq); Pastorino *et al* (1997); Pfeiffer *et al* (1996); Selvaggi *et al* (2002); Shou *et al* (2001); Tateishi *et al* (1994); Yu *et al* (1997); HR>1 implies a worse survival for the group with c-erbB2 expression. The square size is proportional to the number of patients included in the study. The centre of the lozenge gives the combined HR for the meta-analysis and its extremities give the 95% CI.

When considering only the 20 studies evaluating survival in NSCLC, the test of heterogeneity was significant (*P*=0.001). Thus, we calculated the HR using a random-effects model and obtained a value that was statistically significant: HR=1.55 (95% CI: 1.29–1.86) (Figure 1

). When we compared the studies with a quality score above and below the median score (10 studies in each group), we found, respectively, *P*-values of 0.006 and 0.009 for the tests of heterogeneity and HR=1.59 (95% CI: 1.23–2.04) and 1.50 (95% CI: 1.13–2.00).

When we only considered the 17 evaluable studies using immunohistochemistry, there was still heterogeneity (*P*<0.001) and the result of the meta-analysis was statistically significant: HR was 1.46 (95% CI: 1.19–1.78), meaning that patients with a tumour not expressing c-erbB-2 had a better survival rate.

## DISCUSSION

In this systematic review, after aggregation of the available survival results of lung cancer patients according to the tumour expression of c-erbB-2, the presence of this receptor appears to be a prognostic factor for a worse survival in NSCLC.

Some potential important methodological biases need to be discussed. In all, 57% of the evaluable trials were positive compared to 11 % for the nonevaluable ones. There is thus, in terms of publication, a potential bias in favour of the positive trials (overestimation of the magnitude of the true effect of c-erbB-2 overexpression). Out of the 14 significant studies, 12 (86%) are included in the meta-analysis, whereas only nine out of the 16 nonsignificant studies (56%) are included. This could suggest a selection and evaluability bias. This systematic review identified a statistically significant difference in the quality scores between significant and nonsignificant studies evaluating the role of HER-2/neu in lung cancer. The difference in quality score between significant and nonsignificant studies is mainly due to a quality difference in the report of analysis results that are better described in the significant studies. The quality of the design, the description of the laboratory method and the generalisability of the studies were not different, meaning that the quality of the trials for these variables were equivalent in the two groups. This is a known phenomenon: studies with statistically significant results are more likely to be published than those not showing such an effect. The fact of nonpublishing of all nonsignificant data could bias the meta-analysis results in favour of publications with significant results. In order to know if the quality score difference between the significant and nonsignificant studies evaluable for the meta-analysis could distort our results, we compared the studies with a quality score above and below the median score. We found the same results in the two groups: c-erbB-2 could be a bad prognostic factor in NSCLC. The results of the aggregation have, of course, to be considered only exploratory, but could help elaborate statistical considerations for an adequately designed prospective study. If such a study confirmed that c-erbB-2 is a bad prognostic factor for survival, the importance of this prognostic biological factor will probably be small in comparison with other factors such as stage, age or performance status. Indeed, an HR of 1.5 does not mean a large effect of c-erbB-2 on survival and does not constitute a useful factor at the individual level. In our systematic review, out of the 30 articles, eight publications found that c-erbB-2 was a factor of worse prognosis in multivariate analysis.

The techniques used to detect c-erbB-2 expression might also be potential sources of biases. Immunohistochemistry was most frequently used. Immunohistochemical results can vary according to the primary antibody used (Press *et al*, 1994). A number of antibodies were reported in the literature. The dilution of the antibody was sometimes different, leading to problems in comparison among the studies because the sensitivity of the method depends on the antibody concentration. Immunohistochemistry depends on the tissue conservation (fixation in comparison with frozen samples decreases the sensitivity of p185 detection by IHC (Marks *et al*, 1994)) and on the antigen retrieval technique. Moreover, this technique remains mainly qualitative due to some subjectivity in the assessment. The cutoff determining the c-erbB-2 positivity is often arbitrary and varies according to the investigators from a few percent to more than 30% of the tumour cells. The use of different cutoff points is of critical importance (Lee *et al*, 1995). The choice of a cutoff is often arbitrary, although the selection of the median value of the expression levels could be a standard approach to analyse new prognostic factors, even if it may lead to some loss of information. An optimal threshold needs to be defined for c-erbB-2. Lastly, whether membrane or cytoplasmic reactivity should be considered in lung cancer is not clear and the authors assessed c-erbB-2 in different ways.

Therefore, molecular biology techniques are actually necessary in order to validate immunohistochemistry results as already performed in breast cancer. On the other hand, the advantages of immunohistochemistry are the maintenance of the tissue architecture, the ability to localise the antigen and probably a most applicable and cost-effective technique for routine use. c-erbB-2 overexpression can be associated with cleavage of the extracellular domain and its accumulation in the blood (Pupa *et al*, 1993). A good correlation between c-erbB-2 expression in tissue (detected by IHC) and in serum (detected by enzyme immunoassay ELISA) was found in NSCLC (Diez *et al*, 1997) and in adenocarcinoma (Osaki *et al*, 1995), but a lack of correlation between this circulating oncoprotein and tissue expression in NSCLC was found by others (Ardizzoni *et al*, 2001). The inconvenience of ELISA is the requirement of fresh or frozen tissue and calculation of an average c-erbB-2 content of tumour and normal tissue, but the availability for measuring the circulating levels of this protein is an interesting opportunity to assess its overexpression in patients with tumours that are not easily accessible for biopsy. Finally, Hirsch *et al* (2002) found a good correlation between the IHC and FISH results in twothirds of the 51 tumours investigated. Demonstration of HER-2/neu overexpression in NSCLC using a standardised method is essential for establishing clinical trials for anti-HER-2 drugs.

Systematic reviews of the literature are different from meta-analyses of individual patient data. The first approach is only based on fully published studies and provides an exhaustive and critical analysis of the topic with a methodology based on the criteria of Mulrow (1987) and with data aggregation when possible. The second approach is a new study taking into account all performed trials on the topic, published or not, requiring individual data update by the investigators. Our review thus took into account only fully published studies. We did not look for unpublished trials and abstracts because our methodology requires data available in full publications only. In addition, our review deals with prognostic factors studies and, as they are more often retrospective, it is much more difficult to identify unpublished data than for prospective clinical trials data.

As already highlighted in our previous meta-analyses, other potential biases such as language bias or problem in the method of extrapolation of the HR must also be considered (Steels *et al*, 2001; Meert *et al*, 2002a, 2002b).

Why c-erbB-2 overexpression could confer a worse prognosis in NSCLC is not known, especially as it is not related to disease extension and histological subtype. There are some hypotheses with respect to this. c-erbB-2 overexpression regulates cell adhesion and invasive growth of cancer through its association with the cadherin–catenin complex (Ochiai *et al*, 1994) and is associated with increased migratory capacity (Bernstein *et al*, 1994). The coexpression of c-erbB-2 with the EGF-R seems to be more frequent in patients with metastases and seems to be associated with inferior survival (Tateishi *et al*, 1994). Moreover, c-erbB-2 overexpression defined a subgroup of node-negative patients with low angiogenesis and prognosis similar to patients bearing high angiogenesis (Giatromanolaki *et al*, 1996b), supporting the idea that c-erbB-2 expression is a mechanism activated in NSCLC to enable cancer cell migration in tumours with poor vasculature. The potential worse prognosis of NSCLC that express c-erbB-2 is potentially important for prognostic reasons and treatment purposes in addition to improving our understanding of lung cancer biology. Identification of prognostic factors allows defining high-risk groups of patients for whom specific therapy might be necessary, or a stratification has to be performed in controlled trials. Moreover, c-erbB-2 might be a potential therapeutic target and its expression may be linked to a chemoresistant phenotype in NSCLC (Tsai *et al*, 1996). Actually, trastuzumab (Herceptin^R^), a humanised monoclonal antibody that recognises the HER-2/Neu protein receptor, is under investigation for the treatment of lung cancers overexpressing HER-2/Neu, but the choice of the method of detection and the level of Her-2/Neu expression required to obtain a potential therapeutic effect from trastuzumab therapy have not yet been established in lung cancer.

In conclusion, HER-2/neu expression in NSCLC could be a bad prognostic factor in NSCLC, but assessment of c-erbB-2 expression in lung cancer presents several difficulties: the immunohistochemical techniques used to detect its expression and the criteria of positivity varying among institutions. There is, in the literature, a bias favouring the significant studies where the results are described better. Our meta-analysis needs to be confirmed by an adequately designed prospective study in an appropriate multivariate analysis setting taking into account the classical well-defined prognostic factors for lung cancer.
